# Patient-reported quality of life and hand disability in elderly patients after a traumatic hand injury – a retrospective study

**DOI:** 10.1186/s12955-019-1215-9

**Published:** 2019-08-30

**Authors:** Ingrid Reitan, Lars B. Dahlin, Hans-Eric Rosberg

**Affiliations:** 1 Department of Hand Surgery, Skåne University Hospital, Jan Waldenströms gata 5, SE-20502 Malmö, Sweden; 2 Department of Translational Medicine – Hand Surgery, Lund University, Skåne University Hospital, Jan Waldenströms gata 5, SE-20502 Malmö, Sweden

**Keywords:** Elderly, Hand injury, Quality of life, Hand disability, QuickDASH, CISS

## Abstract

**Background:**

Hand injuries occur at any age and cause disability in hand and arm function as well as impaired quality of life, but no study has focused on hand disability and quality of life in the elderly after a hand injury. Globally, the population over 60 years of age is expected to double by 2050 and more hand injuries are estimated among the elderly population. Our goal is to obtain more information and a better understanding of problems elderly patients experience after a hand injury to be able in the future to optimally relocate resources in the health care sector with respect to numbers and injury pattern as well as to health status of these patients.

**Methods:**

Patients aged more than 65 years with a traumatic hand/wrist/forearm injury treated (July 1st 2013 - June 30th 2014) at department of Hand Surgery, Malmö, Sweden were included. Health-related outcome questionnaires, i.e. QuickDASH, SF-36, Visual Analogue Scale (VAS), Cold Intolerance Severity Score (CISS), and general information were mailed to the patients (time from injury: > 1.5–2.5 years). The participants were compared in groups according to age, gender, cold intolerance, injury severity and previous occupation.

**Results:**

One hundred and thirty-seven participants responded [response rate 55%; non-responders (*n* = 113); only difference between groups was that non-responders were older]. Women were older than men at the time of injury (*p* = 0.04) and differed regarding living conditions. The main differences in QuickDASH, all VAS questions, and the majority of SF-36 subscales (*p* < 0.05) were found in the participants with CISS > 50, who experienced more impairment. More serious injuries (Modified HISS) were found to have higher QuickDASH and CISS score as well as more functional impairment (*p* < 0.05). Few differences were found in groups divided according to age, gender (although men experiencing less functional impairment in QuickDASH), previous occupation and injured hand.

**Conclusions:**

Patients aged more than 65 years at the time a hand injury was sustained, generally experience a high-level quality of life and limited functional problems after such an injury, but patients with CISS > 50 and with a more serious injury were more severely affected.

## Background

The health care system has to adapt to global alterations in the pattern of the population that are injured or diseased. In July 2015, the world’s population reached 7.3 billion and the United Nations predicts that it will continue to increase during the coming decades. In Europe, 24% of the population is already over 60 years old, and this is projected to increase to 34% in 2050. Globally, the population over 60 years of age is expected to double by 2050 [1]. This development is placing new demands on health-care systems, due to high numbers of patients with certain conditions, such as cardiovascular diseases and diabetes, and frequent injuries, such as fractures [2–8]. A majority of upper extremity injuries are located in the hand and wrist [9, 10] as well as affected individuals being of any age [10, 11], but any musculoskeletal disorder may occur in the upper extremity [12–14]. Young men usually injure their hands during leisure time and the injuries are minor [10, 15, 16]. Information about hand injuries in the elderly population is lacking, while hand injuries in children have been reported [17–19]. General epidemiological studies of hand injuries show a predominance of women among patients over 65 years of age [10, 16]. The injuries mostly occur during leisure time and are mainly the result of a fall [10, 20].

Few studies have specifically examined function and health in elderly patients with upper extremity trauma. Patients experience various problems after an acute hand injury, including functional limitations, inability to perform physical activities, pain, mood disorders and trauma-related stress [21]. Many of these problems diminish, or are even resolved, during the first year after the injury [21]. However, pain and cold intolerance have been reported as long-standing problems in a ten-year follow-up study in patients after hand trauma [22]. Several studies show that greater problems with the impairment of hand function do not necessarily affect the patients’ activity level or reduce their quality of life [22, 23]. The studies mentioned examined all age groups, but one included no patients aged over 65 years, and the other included only ten retired patients [22, 23].

Evaluation of health-related quality of life and patient reported outcome measurements (PROMS) after injuries are crucial. Health-related quality of life is often measured using self-reported outcome instruments [24]. Patient-reported outcome instruments can measure various aspects of the patient’s health status. This type of data can be accessed from qualitative interviews or questionnaires, where the information comes directly from the patient without interpretation by any mediators. There are a wide variety of questionnaires evaluating patient-reported outcome measurements, ranging from those that ask purely symptomatic questions, including pain and other symptoms (e.g. Disability of Arm Shoulder and Hand; DASH), to those that deal with more complex concepts, such as quality of life, e.g. SF-36 [Short Form-36 Health Survey] [25], but focus has not been on old individuals. Our aim was retrospectively, in an exploratory study, to compare quality of life and functional limitations in groups of elderly patients after a hand injury in order to gain a better understanding of the problems elderly patients experience after this kind of injury.

## Methods

### Participants

This is a retrospective cross-sectional exploratory study using mainly patient-reported questionnaires to collect data. Some additional data were taken from medical charts.

Participants, aged more than 65 years at the time of injury, who were admitted to the Department of Hand Surgery, Malmö University Hospital, in Malmö, Sweden, with a hand/wrist/forearm injury, the latter including the proximal part, between July 1st 2013 and June 30th 2014 were assessed suitable to be included in the study. Distal radius fractures are not treated in the department and only nerves, vessels and tendon injuries as well as extensive skin lacerations in the forearm are treated; thus, patients with a distal radius fractures were not included. The participants were identified (ICD-10 diagnostic system) in another study with a detailed epidemiological evaluation of hand injuries in an elderly population [26], and the same participants were included in the present study. Information concerning age when injured, gender, type of injury, if deceased, suffering from dementia, living abroad, being a foreign-language speaker, and the injured hand were obtained from medical charts in the hospital registry (i.e. no questionnaires sent if dementia, deceased etc.; see below). Participants with a non-injured hand (dominant or non-dominant) were put into groups, while patients who had injured both hands were excluded from these groups (*n* = 6). The participants answered questions concerning status at time of follow up covering; marital status [single/divorced/married/widow(−er)], living situation (together/alone), type of residence (house/apartment/tenancy/sublet), smoking (yes/no), the use of snuff (oral tobacco; yes/no), diseases (yes/no for different groups of diseases), ongoing medication (i.e. none, 1–4, > 4, and total number), current and previous occupation, since these socioeconomic factors and other factors, particularly smoking, may influence the experienced function and health status in a variety of conditions and disorders [27–29]. Occupation was divided into manual work (e.g. craftsmen, lorry drivers, nursing assistants and other types of physical work) and non-manual work (e.g. teachers, shop-assistants and clerical workers).

The Modified Hand Injury Severity Score (MHISS), which is based on the original Hand Injury Severity Score (HISS) [30], were used to classify the severity of the injury, based on information in patient folders (notes and details from surgery) [31]. Depending on the injured structures, a score is obtained. The injuries can then be divided into broad categories, such as “Minor” (≤20), “Moderate” (21–50), “Severe” (51–100) and “Major” (> 100).

Three hundred and ninety-eight patients were treated during the study time. The department treats all hand injuries from the city of Malmö (approx. 427,000 inhabitants, 16% more than 65 years of age) and the city of Lund (approx. 221,000 inhabitants, 15% more than 65 years of age). Patients with a hand injury, requiring specialized hand surgical care, are also referred to the department from the Southern health care region in Sweden (approx. 1,742,000 inhabitants, 19% more than 65 years of age).

The following categories of participants were excluded from the study, based on information from the patient folder system at time of planned mails to the patients (Feb 2016; see below): patients with dementia, those who were deceased at follow up, had sustained a new hand injury, did not speak the Swedish language or were not living in Sweden.

Fifty-six of these patients were injured before the study period (i.e. before July 1st 2013), but were followed up at the department during the inclusion period (i.e. registered in the hospital registry, but not included), 40 did not have a traumatic hand injury according to ICD-10 and 16 patients had only a contusion. In February 2016, when the letters were sent out, some further patients were excluded from receiving mails with questionnaires: 19 patients had died, 13 had dementia, one patient was receiving palliative care, two patients did not speak Swedish and one patient was living abroad.

Two hundred and fifty participants were available for inclusion in the present study. Starting in February 2016 (i.e. > 1.5–2.5 years after the hand injury), letters were sent out to all participants (*n* = 250). These contained written information about the study, a consent form, five different questionnaires [used questionnaires in Swedish and validated [32–34]] and an envelope with pre-paid postage. No relatives or caregivers assisted in completing the questionnaires to our knowledge, but the possibility that a relative or a careprovider assisted in replying the questionnaires cannot be completely ruled out. Only participants who had given their written consent were included in the study.

### Questionnaires

#### Quick disabilities of the arm shoulder and hand (QuickDASH)

The QuickDASH questionnaire (Swedish language) was used to assess present symptoms and physical functioning in the patients [35]. QuickDASH has been shown to have a precision similar to the full length DASH [36] without loss of high reliability and validity [32, 35].

The questionnaire is a region specific instrument and covers daily activities, symptom questions and questions related to self-image and social functioning. The score range from 0 to 100; 0 indicating no disability and 100 signifying the most severe disability.

#### The cold intolerance severity score (CISS)

The CISS questionnaire was originally developed to assess cold intolerance after nerve injuries [37]. Today, it is an instrument used to evaluate cold intolerance after various diseases and injuries [38, 39]. The instrument has been translated into Swedish and retains good reliability and validity [33]. The patients answer six questions regarding present symptoms experienced when exposed to cold. A score of between 4 and 100 is calculated with higher scores indicating more severe cold intolerance [33]. Two groups were analysed; CISS 4–50 and > 50.

#### The short form (36) health survey (SF-36)

The SF-36 questionnaire (Swedish language) is a 36-item generic questionnaire, comprising groups of questions resulting in separate present scores for: physical functioning (PF), role limitations due to physical problems (RP), role limitations due to emotional problems (RE), social functioning (SF), bodily pain (BP), vitality, mental health (MH) and general health (GH). Impaired health status produces low scores on a scale from 0 to 100 [40, 41].

#### Visual analogue scale (VAS)

Six VAS questions (Swedish language), related to present pain, hand mobility, sensory function, grip strength, fine motor skill and sleeping impairment, were sent out. A VAS scale is a 100 mm continuous line with two verbal items in the extremes [42, 43]. A score between 0 and 100 was given; 0 = no pain/impairment to 100 = maximal pain/impairment. The participants were instructed to answer the VAS with respect to the affected hand.

### Statistics

All values presented are median values and the 25th and 75th percentiles. The Kruskal-Wallis and Mann-Whitney U tests were used to compare continuous data. Mann-Whitney U test was used for comparison of two groups. Pearson’s chi-squared test and Fisher’s exact test were used to examine differences in categorical data [presented as n (%)]. If the studied groups tended to be very small they were merged (i.e. MHISS *severe* and *major*) or not included in the analysis (non-injured hand; both *n* = 6). Spearman rank correlation test was used to evaluate correlations between the score of the different questionnaires. A Spearman’s rank correlation coefficient (Spearman’s rho) value > 0.4 was required for significant correlation in order to avoid weak correlations and chosen as a cut point that is clinically meaningful. Effect size was calculated for significant different variables with Eta-squared value (η^2^) with conversion to *d*_Cohen_ and presented as small (0.2–0.4), intermediate (0.5–0.7) or large (> 0.8) effect or the Cramer’s V or phi was computed with interpretation of effect size accordingly. Linear regression analyses were performed to evaluate the effects on the total score of the QuickDASH, adjusted for gender and age, at follow up with focus on relevant factors (i.e. gender, age, CISS, MHISS). The significance threshold was set at *p* < 0.05 in this exploratory study not adjusting for multiple testing [44].

## Results

### Participants

Of two hundred and fifty participants receiving the questionnaires, 102 did not answer, eight participants declined participation, two envelopes were returned as the addresses were unknown and one participant had sustained another injury to the hand (total non-responders; *n* = 113). One hundred thirty-seven participants were included in the study, giving a response rate of 55%. The follow-up time varied from 19 to 40 months depending on when the injury occurred and when the participants returned the questionnaires.

### Non-responders

The non-responders (*n* = 113) were significantly older than the responders (74.0 [69.0–80.5] vs. 70.0 [68.0–75.0] *p* = 0.002). There were no significant differences in MHISS grades (mild/moderate/severe-major for non-responders and responders: 82/24/7 and 87/32/18, respectively; *p* = 0.15) and sex distribution (men/women for non-responders and responders: 48/65 and 73/64, respectively; *p* = 0.10).

### General characteristics and gender

The characteristics of all the participants, also divided into genders, are presented in Tables 1 and 2 with significant differences between men and women concerning age, living situations, marital status, type of residence, and previous occupation (small - intermediate effect size; Table 1), but with no differences regarding use of pharmaceutical drugs or being smokers (most of them non-smokers; Table 2). Men had higher MHISS total scores (intermediate effect size), but did not differ in grades (Table 2). More women had musculoskeletal and rheumatic diseases [*n* = 21 (33%) / *n* = 12 (17%) *p* = 0.044; small effect size].

**Table 1:** Characteristics of 137 patients aged > 65 years after a hand/wrist/forearm injury evaluated 19–40 months after the injury

	All patients (*n* = 137)	Men *n* = 73 (53%)	Women *n* = 64 (47%)	*P*-value^a^
Age at when injured (years) (median[25th–75th percentiles])	70.0[69.0–75.0]	70.0[68.0–72.5]	72.0[69.0–76.0]	0.037
Living situation [alone/together; n(%)]	46(34)/91(66)	10(14)/63(86)	36(56)/28(44)	< 0.001
Marital status [single/divorced/married/widow(−er); n(%)]	8(6)/16(12)/88(64)/25(18)	3(4)/6(8)/60(82)/4(6)	5(8)/10(16)/28(44)/21(33)	< 0.001^c,d^
Type of residence [house/apartment/tenancy/sublet; n(%)]	83(61)/37(27)/16(12)/1(1)	56(77)/10(14)/6(8)/1(1)	27(42)/27(42)/10(16)/0(0)	< 0.001^e,f^
Previous occupation^b^ [non-manual/manual; n(%)]	71(56)/56(44)	32(46)/37(54)	39(67)/19(33)	0.021
Still working? [yes/no; n(%)]	31(23)/106(77)	21(29)/52(71)	10(16)/54(84)	0.101
Occupation if still working^b^ [non-manual/manual; n(%)]	14(47)/16(53)	8(40)/12(60)	6(60)/4(40)	0.442

^a^ = Mann Whitney’s U-test used for continuous data, Pearson’s Chi-Square and Fisher’s Exact test for categorical data. ^b^ = Valid per cent shown due to missing data. ^c^ = Married and widow/−er. ^d^ = Married and divorced. ^e^ = House and apartment. ^f^ = House and tenancy. Values are median and 25th–75th percentiles

**Table 2:** Characteristics of 137 patients aged > 65 years after a hand/wrist/forearm injury evaluated 19–40 months after the injury

	All patients *n* = 137	Men *n* = 73 (53%)	Women *n* = 64 (47%)	*P*-value^a^
Do you smoke?^b^ [yes/no; n(%)]	13(10)/120(90)	9(13)/61(87)	4(6)/59(94)	0.252
Do you use snuff?^b^ [yes/no; n(%)]	6(5)/123(95)	6(9)/62(91)	0(0)/61(100)	0.029
Dominant hand^b^ [right/left/both; n(%)]	111(83)/13(10)/10(7)	60(83)/6(8)/6(8)	51(82)/7(11)/4(7)	0.793
Uninjured hand^b^ [dominant or non-dominant; n(%)]	78(61)/50(39)	43(61)/27(39)	35(60)/23(40)	0.861
MHISS^c^ score (median[25th–75th percentiles])	18.0[6.0–31.0]	20.0[12.0–46.0]	10.0[4.0–27.5]	0.004
MHISS grade [mild/moderate/severe/major; n(%)]	87(64)/32(23)/10(7)/8(6)	43(59)/16(22)/7(10)/7(10)	44(69)/16(25)/3(5)/1(1)	0.136
Number of drugs (median[25th–75th percentiles])	2.0[0.0–4.0]	2.0[0.0–5.0]	2.0[0.0–4.0]	0.669
Number of drugs^b^ [0/1–4/> 4; n(%)]	36(27)/66(49)/33(24)	20(28)/30(42)/22(31)	16(25)/36(57)/11(18)	0.130

^a^ = Mann Whitney U-test used for continuous data, Pearson’s Chi-Square and Fisher’s Exact test for categorical data. ^b^ = Valid per cent shown due to missing data. ^c^ = Modified Hand Injury Severity Score. Values are median [25th–75th percentiles]. *MHISS* Modified Injury Severity Score

### Hand-arm function/symptoms and general health - gender

In Table 3, QuickDASH scores, VAS scales and SF-36 are presented, showing lower scores in SF-36 subscales physical functioning (PF), bodily pain (BP) and mental health (MH) among women (small - intermediate effect size).

**Table 3:** Gender differences in hand-arm function/symptoms and general health after a hand/wrist/forearm injury sustained at an age > 65 evaluated 19–40 months after the injury

	All patients *n* = 137	Men *n* = 73 (53%)	Women *n* = 64 (47%)	*P*-value^a^
QuickDASH^b^ Score	11.4 [2.3–34.1]	8.3[0.6–27.3]	13.6[2.5–42.5]	0.116
VAS^c^ Pain	5.0[2.0–30.8]	5.0[2.0–24.5]	5.5[2.0–37.0]	0.395
VAS Hand Mobility	10.0[3.0–43.0]	11.0[3.0–48.8]	9.0[2.0–39.0]	0.419
VAS Sensory Function	6.5[2.0–36.3]	6.0[2.0–39.3]	6.5[2.0–33.8]	0.998
VAS Grip Strength	15.0[3.0–51.0]	15.0[3.0–54.8.0]	17.5[2.3–49.8]	0.473
VAS Fine Motor Skill	16.0[3.0–54.0]	16.0[3.0–58.0]	15.0[3.0–53.0]	0.995
VAS Sleep	3.0[1.8–20.8]	3.0[1.0–11.8]	3.5[2.0–36.3]	0.090
SF-36^d^ PF^e^	75.0[50.0–95.0]	85.0[65.0–95.0]	70.0[41.3–95.0]	0.013
SF-36 RP^f^	100.0[43.8–100.0]	100.0[25.0–100.0]	100.0[50.0–100.0]	0.796
SF-36 RE^g^	100.0[66.7–100.0]	100.0[66.7–100.0]	100.0[33.3–100.0]	0.781
SF-36 SF^h^	100.0[75.0–100.0]	100.0[75.0–100.0]	100.0[62.5–100.0]	0.322
SF-36 BP^i^	72.0[41.0–100.0]	74.0[46.0–100.0]	52.0[41.0–84.0]	0.041
SF-36 Vitality	70.0[52.5–85.0]	75.0[60.0–87.5]	65.0[45.0–85.0]	0.092
SF-36 MH^j^	84.0[68.0–96.0]	88.0[72.0–96.0]	80.0[65.0–92.0]	0.045
SF-36 GH^k^	72.0[55.5–87.0]	72.0[52.0–91.0]	72.0[55.5–87.0]	0.696
CISS^l^ score	18.0[4.0–35.0]	18.0[6.3–37.3]	14.0[4.0–34.0]	0.423

Presenting median [25th -75th percentiles]. ^a^Mann Whitney’s U-test. ^b^ = Quick Disabilities of the Arm, Shoulder and Hand Score; ^c^ = Visual Analogue Scale; ^d^ = Short Form (36) Health Survey; ^e^ = Physical Functioning; ^f^ = Role Physical; ^g^ = Role Emotional; ^h^ = Social Functioning; ^i^ = Bodily Pain; ^j^ = Mental Health; ^k^ = General Health; ^l^ = Cold Intolerance Severity Score. The score for Quick DASH range from 0 to 100; 0 indicating no disability and 100 signifying the most severe disability. For CISS, a score between 4 and 100 was calculated with higher scores indicating more severe cold intolerance. For SF-36, an impaired health status produces low scores on a scale from 0 to 100. For VAS, a score between 0 and 100 was given; 0 = no pain/impairment to 100 = maximal pain/impairment

### Hand-arm function/symptoms and general health - age

Table 4 shows that participants over 75 years of age had significantly lower scores (small effect size) in SF-36 subscales role emotional (RE), social functioning (SF), general health (GH) and physical functioning (PF).

**Table 4:** Differences in hand-arm function/symptoms and general health after a hand/wrist/forearm injury sustained at an age > 65 evaluated 19–40 months after the injury, divided by age; 66–75 years and > 75 years

	All *n* = 137	66–75 years *n* = 105 (77%)	> 75 years *n* = 32 (23%)	*P*-value^a^
QuickDASH^b^ Score	10.3[2.3–34]	9.1[2.3–29.5]	22.6[2.8–44.9]	0.091
VAS^c^ Pain	5.0[2.0–31.0]	5.0[2.0–26.8]	9.5[3.0–40.3]	0.208
VAS Hand Mobility	10.5[3.0–43.3]	11.0[3.0–43.0]	9.0[2.0–44.8]	0.628
VAS Sensory Function	6.0[2.0–37.0]	9.0[2.0–41.0]	3.0[1.3–21.3]	0.162
VAS Grip Strength	15.0[3.0–51.0]	19.0[3.0–51.0]	10.5[3.0–56.8]	0.821
VAS Fine Motor Skill	15.5[3.0–53.3]	18.0[3.0–53.0]	10.0[2.0–64.8]	0.414
VAS Sleep	3.0[1.5–21.5]	3.0[1.0–19.5]	3.5[2.0–40.8]	0.121
SF-36^d^ PF^e^	77.5[50.0–95.0]	80.0[60.0–95.0]	65.0[36.3–85.0]	0.011
SF-36 RP^f^	100.0[37.5–100.0]	100.0[50.0–100.0]	75.0[25.0–100.0]	0.061
SF-36 RE^g^	100.0[66.7–100.0]	100.0[100.0–100.0]	100.0[33.3–100.0]	0.026
SF-36 SF^h^	100.0[75.0–100.0]	100.0[75.0–100.0]	87.5[62.5–100.0]	0.045
SF-36 BP^i^	72.0[41.0–100.0]	72.0[41.0–100]	52.0[41.0–84.0]	0.420
SF-36 Vitality	70.0[51.3–85.0]	70.0[55.0–85.0]	65.0[40.0–83.8]	0.085
SF-36 MH^j^	84.0[68.0–95.0]	88.0[72.0–96.0]	80.0[60.0–92.0]	0.152
SF-36 GH^k^	72.0[55.0–87.0]	77.0[58.5–90.0]	62.0[40.5–82.0]	0.042
CISS^l^ score	18.0[4.0–35.0]	14.0[14.0–34.0]	20.5[4.0–35.5]	0.537

Presenting median [25th -75th percentiles]. ^a^ = Mann Whitney’s U-test. ^b^ = Quick Disabilities of the Arm, Shoulder and Hand Score; ^c^ = Visual Analogue Scale; ^d^ = Short Form (36) Health Survey; ^e^ = Physical Functioning; ^f^ = Role Physical; ^g^ = Role Emotional; ^h^ = Social Functioning; ^i^ = Bodily Pain; ^j^ = Mental Health; ^k^ = General Health; ^l^ = Cold Intolerance Severity Score. The score for Quick DASH range from 0 to 100; 0 indicating no disability and 100 signifying the most severe disability. For CISS, a score between 4 and 100 was calculated with higher scores indicating more severe cold intolerance. For SF-36, an impaired health status produces low scores on a scale from 0 to 100. For VAS, a score between 0 and 100 was given; 0 = no pain/impairment to 100 = maximal pain/impairment

### Hand-arm function/symptoms and general health - MHISS

Table 5 shows differences between the MHISS grades (*mild, moderate* and *severe-major*) in QuickDASH, CISS, most VAS scales (intermediate - large effect size) and two SF-36 subscales (BP and RP; small - intermediate effect size); mainly found between the *mild* category and the more severe MHISS grades (Table 5).

**Table 5:** Differences in hand-arm function/symptoms and general health in patients after a hand/wrist/forearm injury sustained at an age > 65 evaluated 19–40 months after the injury, divided into groups according to Modified Injury Severity Score (MHISS)

	Mild *n* = 87 (64%)	Moderate *n* = 32 (23%)	Severe-Major *n* = 18 (13%)	*P*-value^a^
QuickDASH^e^ Score	6.8[0.0–21.5]	12.5[6.8–42.0]	35.2[15.9–54.0]	< 0.001^b,c^
VAS^f^ Pain	3.0[2.0–13.0]	24.5[3.3–42.5]	41.0[10.5–64.5]	< 0.001^b,c^
VAS Hand Mobility	5.0[2.0–24.0]	17.0[3.5–43.8]	55.0[26.5–80.0]	< 0.001^b,c,d^
VAS Sensory Function	3.0[2.0–16.0]	17.0[2.0–41.5]	52.0[27.5–78.0]	< 0.001^b,c,d^
VAS Grip Strength	6.0[2.0–41.0]	29.0[3.5–65.0]	55.0[29.0–82.0]	< 0.001^b,c,d^
VAS Fine Motor Skill	6.5[2.0–34.5]	18.0[3.5–52.3]	80.0[46.0–86.0]	< 0.001^b,c,d^
VAS Sleep	3.0[1.0–20.0]	4.0[1.3–36.3]	8.0[2.3–34.5]	0.471
SF-36^g^ PF^h^	80.0[60.0–95.0]	70.0[41.3–95.0]	77.5[32.5–90.0]	0.417
SF-36 RP^i^	100.0[50.0–100.0]	100.0[50.0–100.0]	37.5[0.0–100.0]	0.045^c^
SF-36 RE^j^	100.0[75.0–100.0]	100.0[66.7–100.0]	100.0[0.0–100.0]	0.088
SF-36 SF^k^	100.0[75.0–100.0]	100.0[65.6–100.0]	75.0[46.9–100.0]	0.058
SF-36 BP^l^	74.0[51.0–100.0]	52.0[41.0–84.0]	41.0[22.0–100.0]	0.004^b,c^
SF-36 Vitality	70.0[55.0–85.0]	65.0[51.3–88.8]	67.5[42.5–85.0]	0.766
SF-36 MH^m^	88.0[72.0–96.0]	84.0[69.0–98.0]	78.0[62.0–93.0]	0.602
SF-36 GH^n^	77.0[57.0–90.0]	67.0[50.5–85.8]	68.5[35.0–93.3]	0.611
CISS^o^ score	12.0[4.0–28.3]	25.0[4.0–41.8]	49.0[14.0–64.5]	0.003^c^

Presenting median [25th -75th percentiles]. ^a^ = Kruskal-Wallis test with post hoc tests with Mann Whitney’s U test. ^b^ = mild and moderate; ^c^ = mild and severe-major; ^d^ = moderate and severe-major. ^e^ = Quick Disabilities of the Arm, Shoulder and Hand Score; ^f^ = Visual Analogue Scale; ^g^ = Short Form (36) Health Survey; ^h^ = Physical Functioning; ^i^ = Role Physical; ^j^ = Role Emotional; ^k^ = Social Functioning; ^l^ = Bodily Pain; ^m^ = Mental Health; ^n^ = General Health; ^o^ = Cold Intolerance Severity Score. MHISS = Modified Injury Severity Score. The score for Quick DASH range from 0 to 100; 0 indicating no disability and 100 signifying the most severe disability. For CISS, a score between 4 and 100 was calculated with higher scores indicating more severe cold intolerance. For SF-36, an impaired health status produces low scores on a scale from 0 to 100. For VAS, a score between 0 and 100 was given; 0 = no pain/impairment to 100 = maximal pain/impairment

### Hand-arm function/symptoms and general health - high or low CISS

Table 6 shows that participants with CISS > 50 scored significantly higher on QuickDASH (large effect size), all VAS (intermediate - large effect size), and MHISS total score (intermediate effect size), while lower scores were found in all SF-36 subscales (small - intermediate effect size), except for GH and vitality.

**Table 6:** Differences in hand-arm function/symptoms and general health in patients after a hand/wrist/forearm injury sustained at an age > 65 evaluated 19–40 months after the injury, groups divided according to high or low cold intolerance severity scores (CISS)

	CISS 4–50 *n* = 113 (86%)	CISS > 50 *n* = 18 (14%)	*P*-value^a^
Age	70.0[68.5–75.0]	71.5[68.0–76.3]	0.936
QuickDASH^b^ Score	7.5[0.6–22.8]	51.1[35.8–71.0]	< 0.001
VAS^c^ Pain	4.0[2.0–19.5]	49.5[8.3–72.0]	< 0.001
VAS Hand Mobility	8.0[2.0–34.0]	43.0[11.0–71.5]	0.001
VAS Sensory Function	3.0[2.0–23]	50.5[27.5–79.5]	< 0.001
VAS Grip Strength	7.5[2.3–45.0]	72.5[20.3–82.0]	< 0.001
VAS Fine Motor Skill	10.0[3.0–45.0]	76.5[34.3–88.3]	< 0.001
VAS Sleep	3.0[1.0–14.0]	16.5[3.5–44.5]	0.022
SF-36^d^ PF^e^	85.0[60.0–95.0]	50.0[25.0–81.3]	0.001
SF-36 RP^f^	100.0[50.0–100.0]	50.0[0.0–100.0]	0.016
SF-36 RE^g^	100.0[100.0–100.0]	66.7[16.7–100.0]	0.004
SF-36 SF^h^	100.0[75.0–100.0]	75.0[46.9–100.0]	0.001
SF-36 BP^i^	74.0[43.5–100.0]	41.0[28.8–67.5]	0.007
SF-36 Vitality	70.0[55.0–85.0]	65.0[28.8–77.5]	0.085
SF-36 MH^j^	88.0[72.0–96.0]	72.0[62.0–81.0]	0.006
SF-36 GH^k^	77.0[57.0–90.0]	69.5[32.5–82.5]	0.285
MHISS^l^ total score	16.0[5.0–30.0]	29.0[18.0–89.3]	0.004

Presenting median [25th -75th percentiles]. Six missing items of data. ^a^ = Mann Whitney’s U-test continuous data, Fisher’s Exact test for categorical data. ^b^ = Quick Disabilities of the Arm, Shoulder and Hand Score; ^c^ = Visual Analogue Scale; ^d^ = Short Form (36) Health Survey; ^e^ = Physical Functioning; ^f^ = Role Physical; ^g^ = Role Emotional; ^h^ = Social Functioning; ^i^ = Bodily Pain; ^j^ = Mental Health; ^k^ = General Health; ^l^ = Modified Hand Injury Severity Score. MHISS = Modified Injury Severity Score. The score for Quick DASH range from 0 to 100; 0 indicating no disability and 100 signifying the most severe disability. For SF-36, an impaired health status produces low scores on a scale from 0 to 100. For VAS, a score between 0 and 100 was given; 0 = no pain/impairment to 100 = maximal pain/impairment

### Hand-arm function/symptoms and general health - previous occupation

Participants with an earlier manual occupation had lower scores on several of the SF-36 subscales (small – intermediate effect size; Table 7).

**Table 7:** Differences in hand-arm function/symptoms and general health in patients after a hand/wrist/forearm injury sustained at an age > 65 evaluated 19–40 months after the injury, divided into groups according to previous occupation (manual/not-manual)

	Manual *n* = 56 (44%)	Non-manual *n* = 71 (56%)	*P*-value^a^
QuickDASH^b^ Score	18.2[2.3–37.5]	9.1[0.0–25.0]	0.072
VAS^c^ Pain	5.0[2.0–41]	4.0[2.0–30.0]	0.439
VAS Hand Mobility	12.0[3.0–44.0]	8.0[2.0–43.0]	0.261
VAS Sensory Function	9.0[2.0–40.0]	5.0[1.0–33.0]	0.408
VAS Grip Strength	17.0[4.0–73.0]	21.0[3.0–46.0]	0.176
VAS Fine Motor Skill	19.0[2.0–55.8]	13.0[3.0–55.0]	0.675
VAS Sleep	4.0[2.0–29.0]	3.0[1.0–19.5]	0.245
SF-36^d^ PF^e^	70.0[46.3–85.0]	85.0[60.0–95.0]	0.003
SF-36 RP^f^	75.0[25.0–100.0]	100.0[75.0–100.0]	0.001
SF-36 RE^g^	100.0[33.3–100.0]	100.0[100.0–100.0]	0.043
SF-36 SF^h^	87.5[75.0–100.0]	100.0[84.4–100.0]	0.040
SF-36 BP^i^	62.0[41.0–100.0]	74.0[48.5–100.0]	0.299
SF-36 Vitality	65.0[50.0–78.8]	75.0[55.0–90.0]	0.033
SF-36 MH^j^	84.0[72.0–92.0]	92.0[68.0–96.0]	0.324
SF-36 GH^k^	67.0[41.3–87.0]	77.0[57.0–92.0]	0.129
CISS^l^ score	18.0[4.0–35.0]	17.0[4.0–33.8]	0.726

Presenting median [25th -75th percentiles]. Ten items of data missing. ^a^ = Mann Whitney’s U-test used. ^b^ = Quick Disabilities of the Arm, Shoulder and Hand Score; ^c^ = Visual Analogue Scale; ^d^ = Short Form (36) Health Survey; ^e^ = Physical Functioning; ^f^ = Role Physical; ^g^ = Role Emotional; ^h^ = Social Functioning; ^i^ = Bodily Pain; ^j^ = Mental Health; ^k^ = General Health; ^l^ = Cold Intolerance Severity Score. The score for Quick DASH range from 0 to 100; 0 indicating no disability and 100 signifying the most severe disability. For CISS, a score between 4 and 100 was calculated with higher scores indicating more severe cold intolerance. For SF-36, an impaired health status produces low scores on a scale from 0 to 100. For VAS, a score between 0 and 100 was given; 0 = no pain/impairment to 100 = maximal pain/impairment

### Hand function - dominant or non-dominant

VAS sensory function and grip strength (i.e. lower VAS value) were better in participants who did injure their non-dominant hand compared with those who did injure their dominant hand (sensory function; 3.0[1.0–13.5] and 12.0[2.0–42.7], respectively, *p* = 0.022; grip strength; 5.0[2.0–44.0] and 27.5[3.8–60.3], respectively, *p* = 0.043; small - intermediate effect size). No other differences were found in the other variables (results not shown).

### Correlations between scores from questionnaires

Generally, there was a positive correlation between the QuickDASH and VAS questionnaires (i.e. pain, range of motion, sensibility, grip strength, fine dexterity; rho-values between 0.55–0.73; *p* < 0.0001) and with CISS score (rho-value = 0.59; p < 0.0001). Furthermore, there was generally a negative correlation (rho-values = − 0.40 - -0,67; p < 0.0001) with the eight subcomponents of SF-36 and the QuickDASH score.

The VAS questions positively correlated between each other (rho-values = 0.62–0.74; *p* = 0.0001), but these questions did not correlate with SF-36 (except bodily pain with VAS pain rho-value = − 0.58; *p* = 0.0001).

CISS score positively correlated with the VAS questions (rho-values = 0.47–0.60; p = 0.0001), but not with SF-36.

### Linear regression analysis

In two different linear regression models, the effects of different factors on the total score of QuickDASH were analysed (adjusted for gender and age). Male gender (unstandardized B-coefficient − 6.3, 95% CI -12.4 - -0.2; *p* = 0.041) and the value of the CISS total score (unstandardized B-coefficient 0.66, 95% CI 0.52–0.80; p = 0.0001) were associated with the QuickDASH total score at follow up; thus, male gender lowered the QuickDASH score and a higher value of the CISS score increased the QuickDASH score at follow up. There was no association between age (*p* = 0.14) or the MHISS score (*p* = 0.38) with QuickDASH total scores at follow up. By introduction of dummy variables for grade of MHISS and group of CISS and performing the regression analysis, male gender (unstandardized B-coefficient − 6.9, 95% CI -13.5- -0.3; *p* = 0.042), a CISS score > 50 (unstandardized B-coefficient 31.7, 95% CI 22.2–41.2; *p* = 0.0001) as well as moderate (unstandardized B-coefficient 8.1, 95% CI 0.5–15.7; *p* = 0.038) and severe/major (unstandardized B-coefficient 16.2, 95% CI 6.0–26,5; *p* = 0.002) grade of MHISS were associated with the QuickDASH total score at follow up; thus, male gender lowered the QuickDASH score, while a CISS score> 50 as well as moderate and severe/major MHISS grades increased the QuickDASH score at follow up.

## Discussion

The present retrospective exploratory study has shown differences in hand disability and quality of life in elderly participants with an age more than 65 years, who sustained a hand injury more than 1.5 years earlier, grouped by variables, such as gender, age, severity of injury, cold intolerance and injured hand. The most obvious differences were found in participants with abnormal cold intolerance (i.e. CISS > 50) that experienced impaired quality of life (small – intermediate effect size) and hand function (large effect size); e.g. expressed as an association in regression analyses with CISS score or CISS group with an increased QuickDASH score at follow up. No existing studies have highlighted these aspects based on a variety of mailed questionnaires showing various aspects of health and disability.

Men and women differed regarding their living situation, reflecting the current general elderly population in Sweden [45]. However, few differences were found in hand disabilities and general health, where women experienced some limitations in physical functioning and had more bodily pain (small – intermediate effect size). They were found to be older than the men at the time of the injury, and had more musculoskeletal and rheumatic diseases, which may to some extent explain the differences observed; e.g. observed as the association between male gender having a lower QuickDASH score at follow up, while age did not influence the total QuickDASH score in the regression analyses. The oldest participants appeared to function as well as the younger regarding their hand and arm, and differed only in half of the SF-36 parameters (i.e. physical functioning, emotional role, social functioning and general health; small effect size). However, self-reported effects on general health and physical functioning might be expected in the oldest participants considering their aging bodies. Physical disabilities and social issues are well known complications in older patients [46, 47].

The *severe* and *major* groups of MHISS grades were merged since these two groups had fewer participants and involve quite advanced injuries; both of them being complicated [30]. Men generally had higher MHISS total scores, but not so high that the MHISS grades (i.e. *mild, moderate and severe-major*) differed between the genders. Several differences in QuickDASH, VAS and CISS (intermediate – large effect size) were found within the severe-major MHISS groups compared with *mild* injuries, but almost no differences were found in the quality of life domains (SF-36; small effect size). However, moderate and severe-major grades of MHISS were associated with substantially higher total QuickDASH scores at follow up as observed in the regression analyses. The findings in QuickDASH and other quality of life variables indicate that even if there is a disability in hand function, also elderly people adapt and learn to cope with the injury and any residual problems to the extent that it does not influence their quality of life [22, 23, 48]; i.e. the “well-being paradox”, where objectively negative factors in the patient’s life have relatively little effect on their perceived quality of life [49].

Cold intolerance appears for unknown reasons following all types of hand injuries [37, 50, 51]. Cold intolerance was found to be the variable that had the most impact on quality of life (small – intermediate effect size) and particularly hand function (large effect size). The participants with abnormal CISS scores, i.e. CISS score > 50 indicating abnormal cold intolerance [52], reported worse impairment observed in almost every questionnaire, particularly concerning hand and arm function. The CISS score also positively correlated with QuickDASH and the VAS questionnaires, indicating the complexity of hand injury and disability. In agreement with the present finding, an association between cold intolerance and higher QuickDASH scores [39, 53], impaired, self-reported, quality of life in SF-36 [33] as well as severity of the injury has also previously been reported [52]. Cold sensitivity is one of the worst symptoms experienced by patients with a post-traumatic hand injury, also being the most limiting symptom regarding daily life [54, 55]. Patients with high CISS scores should be informed as early as possible about strategies for relief [54] in order to improve both quality of life and hand function.

Participants, who previously had a manual occupation, had lower scores in some SF-36 subscales (small – intermediate effect size). In a study examining quality of life, performed 1 year after a hand injury, results suggest that “blue-collar workers experience functional limitation to a greater extent than white-collar workers” [21]. These patients had an ongoing work situation, and one could assume that, as they used their hands more on a daily basis, they experienced a worse life situation [21]. Our study, evaluating the participants presently reported situation, may also indicate a consequence (such as reported pain problems due to other conditions, e.g. osteoarthritis) of an earlier physical work situation in their previous professional life.

Few differences (sensory function and grip strength; small - intermediate effect size) were found concerning whether the participants had injured the non-dominant or dominant hand for which it is difficult to find a logical explanation. In view of the present follow-up time (i.e. 19–40 month) and considering that most of the participants had *mild* injuries according to MHISS, there might have been no long-lasting impairments to the injured hand. This kind of analysis might be more valuable with a shorter follow-up time, while the injured hand is still affected [21].

### Strengths and limitations

The major limitation of the study is its retrospective and cross-sectional nature. The response rate (55%) accords well with the mean response rates in articles published in medical journals (60%) [56]. The non-responders were older than the responders without any differences in gender or MHISS grades; thus, the responders being presumed to be representative of the studied group, although we cannot completely rule out that the non-responders had more extensive dysfunction or disability due to a slightly higher age. The patient related outcome measures used, i.e. QuickDASH, SF-36 and CISS, are validated instruments in studies relevant for the present study and were used in Swedish language [32, 33, 40, 41]. However, the specific used VAS questions have not been validated in Sweden for this patient group, why one should consider the known limitations of using VAS questions, but still the obtained present information add value how the participants experience their symptoms, function, and life quality (i.e. sleep). We did not perform more complex statistical analyses of the present population due to its retrospective nature of the study, but the present study generates new hypotheses for further research concerning hand injuries in the elderly patients.

## Conclusions

Bearing in mind that the world can expect an increasing number of physically active elderly people, more careful investigation of this group of patients is relevant. Few differences in quality of life, assessed in groups by gender, age, previous occupation and injured hand were found. In general, the participants felt they had a good quality of life and few daily limitations, although hand function, evaluated by QuickDASH, were influenced by female gender and severity of the hand injury. More important, participants with abnormal cold intolerance (i.e. CISS > 50) experienced particularly impaired hand function, but also some reduced quality of life. Gaining a better knowledge in prospective studies about the health, as well as the complex and integrated hand function, of geriatric patients after hand injuries could lead to a better evaluation of ongoing clinical practice and provide an opportunity for improving quality of care. Furthermore, information about any impairment of function and health after injuries and diseases in an elderly population may give us a tool for possibilities to (re-)allocate resources within the health care sector and other sectors for such elderly patients with injuries that may substantially increase in numbers in the future.

## Data Availability

Public access to the data is restricted by the Swedish Authorities (Public Access to Information and Secrecy Act; http://www.government.se/information-material/ 2009/09/public-access-to-information-and-secrecy-act/), but data can be made available for researchers after a special review that includes approval of the research project by both an Ethics Committee and the authorities’ data safety committees.

## Abbreviations

BP: Bodily Pain (SF-36)
CISS: Cold Intolerance Severity Score
GH: General Health (SF-36)
HISS: Hand Injury Severity Score
MH: Mental Health (SF-36)
MHISS: Modified Hand Injury Severity Score
PF: Physical Functioning (SF-36)
QuickDASH: Quick Disabilities of the Arm Shoulder and Hand
RE: Role Emotional (SF-36)
RP: Role Physical (SF-36)
SF: Social Functioning (SF-36)
SF-36: The Short Form (36) Health Survey
VAS: Visual Analogue Scale
